# Supplementary material for: Extracting temporal relationships between weakly coupled peptidergic and motoneuronal signaling: Application to Drosophila ecdysis behavior
Source: PLoS Comput Biol. 2021 Dec 15;17(12):e1008933. doi: 10.1371/journal.pcbi.1008933 (PMC8716061; doi:10.1371/journal.pcbi.1008933)
Supplement: S1 Fig — (a) Projection of 5 images from different planes, of GCaMP3.2-expressing CCAP neurons and motoneurons. (b) Time series for each motoneuron and average for each side. (c) Correlation matrix of the motoneuron activity traces, showing the highest correlation between motoneurons of the same side. (PDF) [file pcbi.1008933.s001.pdf]

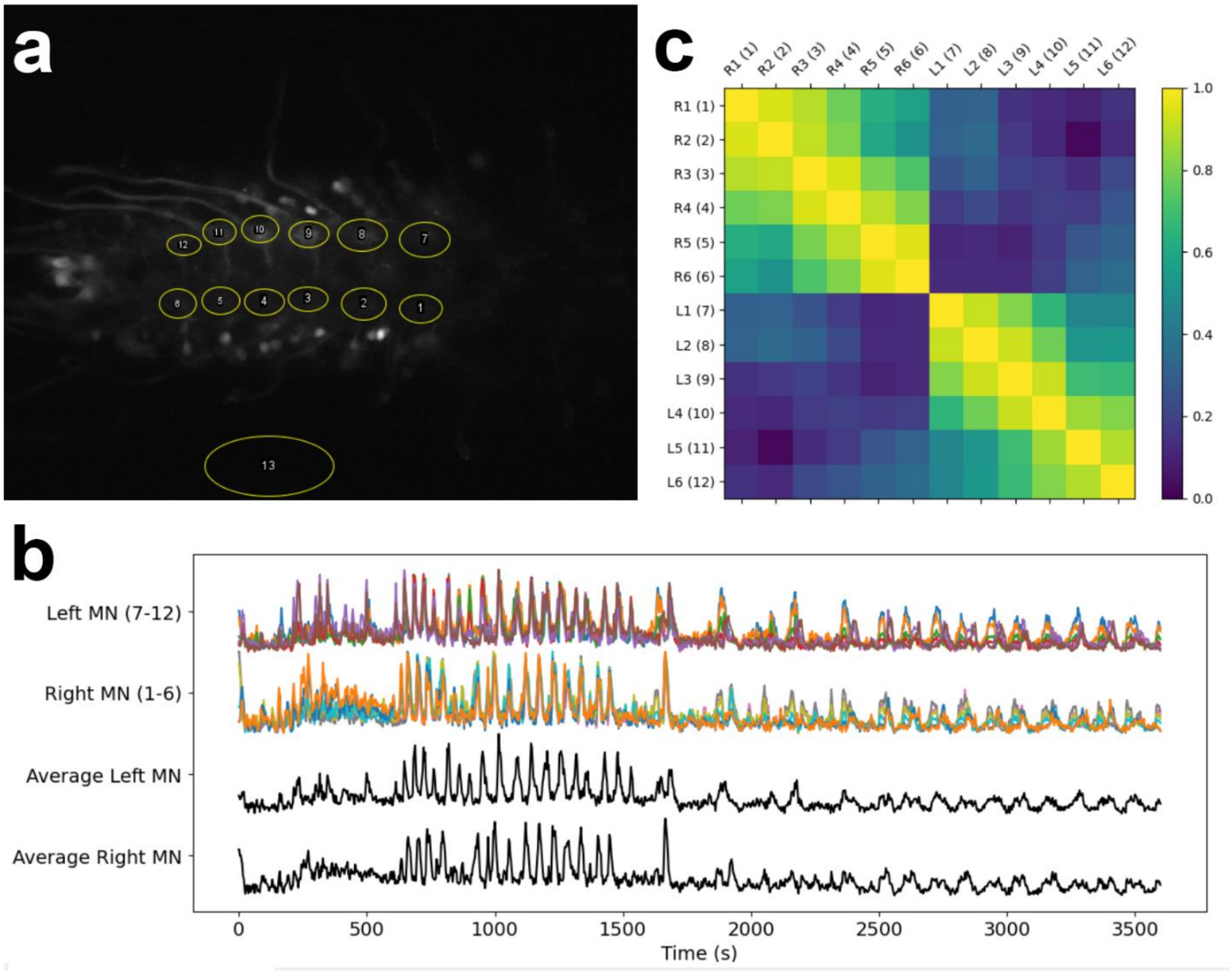

S1 Figure. Motoneuron coordination

(a) Projection of 5 images from different planes, of GCaMP3.2-expressing CCAP neurons and motoneurons. Numbered regions are the quantified ROIs: 6 left motoneurons (7-12), 6 right motoneurons (1-6) and a background region (13). (b) Fluorescence time series for each motoneuron and average corresponding to each side. Time series are generated through the division of the raw motoneuron fluorescence time series by the background fluorescence time series. (c) Correlation matrix of the motoneuron activity traces, showing the highest correlation between motoneurons of the same side. Note that when the whole time trace is considered, no negative correlation is found.
