# Supplementary material for: Extracting temporal relationships between weakly coupled peptidergic and motoneuronal signaling: Application to Drosophila ecdysis behavior
Source: PLoS Comput Biol. 2021 Dec 15;17(12):e1008933. doi: 10.1371/journal.pcbi.1008933 (PMC8716061; doi:10.1371/journal.pcbi.1008933)
Supplement: Attachment — Submitted filename: AnswerToReviewers.docx [file pcbi.1008933.s003.docx]

**Reviewer #1: This manuscript considers how the temporal aspects of neuromodulation are involved in the production of behavior. Specifically, the authors seek to address the role of temporal modulation in the control of Drosophila ecdysis by describing the influence of peptidergic modulation from CCAP-releasing neurons on motor neurons. Towards this, the authors simultaneously measure the population activities of CCAP and motor neurons during fictive ecdysis behavior in ex-vivo preparations using calcium imaging, and report that the peptidergic neuron population activity is tightly coupled to the activity of the faster motor neuron population. Computational models are constructed to capture the calcium dynamics of CCAP neurons, and simulations demonstrate that these models mostly recapitulate experimental observations. Further, the authors develop an algorithm to classify the stages of in-vivo ecdysis behavior. These stages are then compared to ex-vivo motor neuron activity.**

**The significance of the work is the attempt to link the influence of peptidergic modulatory neuron activity on motor neurons with behavior.**

**Major Comments**

**The manuscript is missing key information about methodology that severely limits the interpretation of the results.**

**The calculation of activity onset doesn’t appear to be defined in the manuscript.**

*R: We understand the concern of the reviewer, but this information was given in the Methods section, under the sub-title ‘Activity Onset’ (line 544, now 557). Perhaps it was missed or overlooked for being at the end of the page. In the updated version, this is also mentioned in the Results section (line 123-124)*

**Figure 1 c suggests that the signal of L and R motor neurons are being grouped.**

**The claim that there are no major differences in the activity between sides is not supported in the text. Please include a reference or a panel that supports this point.**

**Why are L and R motor neurons being grouped? The analysis should work with the individual motoneuron data points.**

*R: We thank the reviewer for this observation. We have clarified this issue in the text (lines 116-118), which now reads….”* *Regarding motoneurons, we detected that the activity on each side of the animal is highly correlated (Figure S1). Thus, we divided them into left and right regions, as there were no major differences in the activity between sides and calculated the average motoneuronal activity of each side”. In addition, we have included a supplemental figure that shows some examples of activity of individual motoneurons and how neurons from the same side are highly correlated if not identical. In fact, if this were not the case, we would not* *have obtained such a clear oscillation after grouping, but rather an incoherent and noisy time series.*

**Also, the one-tailed Mann-Whitney U test is sensitive to the variance of the data which would be under-represented when averaging the L and R motor neurons.**

*R: The Mann-Whitney test was employed to compare features extracted from the time series, such as onset and duration of the oscillations. Therefore, we think that the reduction in the variability in the time series due to averaging is unlikely to interfere with this analysis. Moreover, we now show in S1 Figure that the time series being averaged are very similar during the period of oscillatory behavior, and thus that the variance is expected to be low.*

**The modeling component of this work is interesting but does not contribute to any point made in the manuscript. The authors should better explain the rationale for the models. Mainly, addressing the utility of the ability to predict motor neuron activity from modeled CCAP activity using ad hoc tuned models.**

*R: We agree with the reviewer in that we did not explain properly the rationale behind the models. The first model (statistical model) was used to show that MN activity could be predicted based on the pattern of CCAP activity. As we detail in our response to Reviewer 2, below, this is a step forward compared to simply observing correlations between activities, because it links more tightly the respective time traces. The second model is a first step towards a mechanistic interpretation of the oscillatory behavior of MNs. Although many elements are still missing in order to provide a meaningful model, future work can take it as a first step and also incorporate new experimental evidence as it becomes available. We have added text at the beginning of the first model to emphasize this. Also, in the discussion the following text can be found: “Our analysis methods provide a framework to interpret new experimental manipulations that can be made to the mechanisms downstream of the CCAP command signal.”*

**Maybe these models are useful to predict behavioral state transitions? If true, this should be highlighted and so far isn’t clear in the text.**

*R: The reviewer is correct that this is what the first model does: it predicts the probability of MN oscillatory behavior and thus can predict the start of ecdysis. However, at present we can only predict the activity of MNs in an ex-vivo preparation, and we cannot be sure that the activity of the whole animal will be similar. This is certainly a direction in which we would like to progress, but at this time it seems far from feasible. The main reason is simple: it* *requires being able to record the activity of CCAP neurons in an intact animal,* *which is currently not possible.*

**ln 414: Why is there an attempt to classify motor neuron calcium recording using the results of the behavior algorithm?**

*R: Perhaps we do not understand what the reviewer means by ‘classify’, but the intention of this part of the manuscript was just to compare calcium recordings with the quantification of behavior. The aim of this was to see to what extent these two measurements were correlated, under the assumption that motor commands are all that is needed to explain movements (the results show that the assumption is wrong at least in a quantitative sense).*

**Behavior is an output that integrates multiple signals, and is usually a nonlinear function of motor neuron output. Variability is expected to decrease from motor neuron activity to behavior output simply because there are fewer degrees of freedom in the biomechanics.**

*R: We agree that behavior depends on many variables and at the same time possesses fewer degrees of freedom than do the patterns of motor neuron activity. Indeed, in this work we have been able to show that the degrees of freedom of motor neuron activity can be reduced by averaging the activity from the left and right sides, and by expressing the activity as the difference between the activity of both sides. Nevertheless, our proposal that the oscillation of behavior is more regular than that of MN activity because of sensory feedback is nothing more than a plausible hypothesis. To test it, recordings of MN activity would have to be done in the whole moving animal, something that is not possible at the moment. Of course, other factors such as the biomechanics of movements in the pupa also play a role, and we have added a note in this effect in lines 505-506.*

**The finding about post-ecdysis phase being divided into fast and slow contraction frequencies as currently presented in the results and discussion (ln 495-500) does not contribute to the apparent main point of the paper (determining the interactions between peptidergic modulation and production of behavior)**

**Suggest to either better integrate for clarity or remove to prevent diluting the focus of the manuscript.**

*R: We agree with the reviewer in that the newly found post-ecdysis phase is not in the line of the main study presented. However, it is a novel finding that we found worth noting. We have now added a brief remark in order to provide some context to this finding.*

**Minor Comments**

**Line 65-66: “Ecdysis begins with the release of Ecdysis Triggering Hormone (ETH) into the circulatory system (hemolymph)...”**

*R: Thank you for noting this, it has been fixed.*

**Line 78: The methods listed (PCA, ICA, SVD, k-means) are thought to be fairly general. What is meant by “restricted to specific datasets”?**

*R: We have rewritten this sentence for clarity.*

**Line 79: Offering more specific reasons for the introduction of new methods would strengthen the argument. See previous point.**

*R: Thank you for the suggestion, this sentence has been rewritten.*

**Line 80: It’s not clear what is new about the computational approaches -- many of these approaches have been around for some time.**

*R: We agree; the approach presented is not new, so we have removed ‘new’. Nevertheless, we should point out that these computational approaches are new to the field of ecdysis.*

**Line 87: “... simulates many of the observed experimental features.”**

*Fixed*

**Line 93: Replace “through” with “throughout”**

*Fixed*

**Line 116 - 118: Unclear what is being grouped (117), when motoneurons are being divided into left and right regions.**

*R: We apologize for the lack of clarity. What we did was to define motoneuronal activity as the average of several ROIs for the right (or left) region. We have clarified this better in the text and S1 Figure clarifies this further*.

**Line 120: Perhaps this is a term that I’m unfamiliar with, but unsure what is meant by “ecdysial phase proper”**

*We agree that our writing lacked clarity. We have now substituted the entire sentence by “… the onset of left-right alternating motoneuronal activity corresponds to the beginning of the ecdysial phase of the ecdysis motor sequence, after the pre-ecdysis phase has finished”.*

**Line 123: What do the +/- indicate? Variance, SD, SE?**

*R: We apologize for the missing information. It is SE, standard error of the mean. The information has now been added to the corresponding text and figure legends.*

**Lin 128: While technically true, not a completely fair statement since figure 1c shows 4 out of 9 experiments demonstrating the opposite.**

*R: The reviewer may be referring to Figure 1d. If one only looks at the averages, then it may indeed appear that the opposite statement may more accurately describe our results. However, when looking at the individual points, the onset of activity for MNs is almost always (with one exception) preceded by the onset of activity of at least one CCAP neuron (usually more than one). We have made this clearer in the text, which now reads “…the onset of motoneuron activity usually starts after at least two or three CCAP neurons, …”.*

**Line 150: “In contrast” instead of “By contrast”**

*Fixed*

**Line 368: Repeated “Fig 9”**

*Fixed*

**There is little discussion about how the activity of other modulatory neurons may be involved. This is not a deal-breaker, but what is known should be presented to better frame the results (ex. What other modulatory projections exist, how might that influence the interpretation of results)**

*R: We appreciate the reviewer’s suggestion. As mentioned in the introduction of the manuscript, there are several modulators including neuropeptides that are involved in the generation of ecdysis behavior. Indeed, we have previously shown that removing any of the key neuropeptides from the ecdysial neural system (such EH, PBURS or ETH) causes significant changes to the pattern of activity of CCAP neurons and motoneurons as well as to the ensuing behavior (Mena et al., 2016). In the future, we could complement the results presented here by using our analysis pipeline in simultaneous CCAP and motoneuronal recording carried out in mutant flies lacking one of these peptides. In particular, it would be interesting to study the role of neuropeptide EH, because we have previously reported that a lack of EH causes a significant alteration to the temporal organization of the activity of CCAP neurons (Mena et al., 2016). Therefore, our quantitative approach could help to characterize the role of EH and of other neuropeptides in coordinating the temporal relationships between CCAP neurons and their relationship with the resulting rhythmic motor outputs. A brief paragraph with these ideas has been added to the discussion.*

**Figure 1:**

**1a and 1b are missing scale bars**

*R: Figure 1a now includes a scale bar, which applies to 1b as well. Also, Figure 1b has been replaced by one from the same experiment as the one shown in Figure 1a.*

**1d:**

**It is not clear what the vertical bars indicate. May be more informative to represent this data as a box and whiskers plot.**

*R: We agree with the reviewer’s suggestion; bar plots in Figures 1,2,5 and 11 have now been replaced by box plots with overlaid individual data points.*

**Please indicate precisely what is meant by “onset” -- a simple schematic or indication on 1c would help.**

*R: We have added a small arrow to Figure 1C to clarify this point.*

**Reviewer #2: I consider that this paper contains significant novelty in the context of the study of the relationship between slow dynamics of neuromodularoty neurons and motoneurons and delivers both experimental and mixed experimental and modeling results that contribute to understand this relationship. Thus, in my opinion the paper is suitable for publication in PLoS Computational Biology. However, I find several issues that could be addressed to improve the paper:**

*We were pleased to read that the reviewer found our paper of interest and suitable for PLoS Computational Biology. We have done our best to improve the paper by responding to their comments.*

**1-It is clear from the timescales shown in the figures that the correlation analysis focuses on slow dynamics. Authors could comment on the slow time scale nature of the calcium imaging of both motoneurons and CCAP neurons. Is this the only relevant time scale of the interaction? Please discuss if motoneuron voltage activity is known to faster than calcium dynamics.**

*R: As the reviewer suggests, the membrane voltage of motoneurons can change much more rapidly than does the calcium dynamics. However, the firing rate of MNs, which is the relevant output signal for the activity of muscles, is very well approximated by the time course of the changes in intracellular calcium levels. High firing rates cause the entry of calcium through voltage-activated calcium channels and when firing ceases the ion is rapidly buffered or extruded from the cell.*

**2-Please specify the number of recordings for each experimental trial in the statistics shown in Fig. 1d.**

*R: We apologize for the lack of clarity. In Figure 1d, each couple of bars corresponds to a single experiment, while each dot represents either a single cell (in the case of CCAP) or either the left or right side of the nervous system (for motoneurons). We have clarified these details in the Figure legend, and bars have been replaced by box plots.*

**3-Please explain how did you group the neurons to increase the signal to noise ratio.**

*R: To characterize the activity of motoneurons we averaged several ROIs from either the right or the left side of the animal, as described in the newly supplied S1 Figure. Then, and as described in Methods, the brightness intensity in each ROI was averaged and normalized accordingly. We have now clarified these details in the text.*

**4-The paper is focused on the frequency analysis, but regarding the coordination of motor movements it would be more useful an analysis of the sequences that built up the motor program (beyond the phase analysis), i.e., what is the effect of the neuromodulation in the sequential activation of the motoneurons that shape the behavioral observations. This could be at least discussed at the end of the manuscript, as a limitation of the proposed analysis and modeling approach if it cannot be addressed.**

*R: The reviewer raises a concern that we share. As we mention in the manuscript, very little is known about the neurons involved in producing the motor program sequence –other than the motoneurons themselves. Thus, there are few clues about which genetic markers could be used to tag them with fluorescent calcium probes and follow their activity in real time. This would constitute a whole different study, in which the methodology presented here could be used as a tool for analyzing and interpreting the data.*

**5-It is not very surprising that the logistic model captures the correlation from the model design.**

*R: We agree with the reviewer that the result is in principle not very surprising. Nevertheless, it worth pointing out that correlation measurements consider the entire trace, whereas the logistic model (or the conductance-based model) may consider other subtleties in the time course. Moreover, both models can easily be expanded into a model with ‘memory’, in which the probability of oscillation depends not only on CCAP activity at any given time but also on its past activity. We did not pursue this idea because the simple model provided satisfactory results*

**6-How could the proposed methodology be used in other experimental models. This could be important to target a general readership.**

*R: We appreciate the reviewer’s suggestion. Our analysis could easily be applied to any type of experimental data that involves the coordinated activity of several neurons or groups of neurons that can be independently recorded. This includes, but it is not restricted to, recordings in the stomatogastric ganglion of the lobster, the segmental ganglia of the leech, spinal cord in lamprey, Aplysia buccal ganglion or abdominal segments in Manduca sexta and Drosophila melanogaster. In many of these organisms, the circuits that generate oscillatory behavior are mainly known, but there are still gaps that could be filled using genetic or pharmacological manipulations, combined with the analysis we present here. We have now included these ideas in the discussion.*

**Minor issues:**

**6-Why is real-time calcium-imaging mentioned in the abstract. Why is the real-time nature of the calcium imaging important. I think this can be misleading for some readers.**

*R: This is indeed misleading and unnecessary. The expression was removed.*

**7-There are several problems with the dynamic references to the figures, e.g. lines 237, 254, 274, 291, 317, 335, 404, 415.**

*We apologize for this error. Apparently, the error originated during the automatic compilation by the submission system. To avoid this problem, we have now uploaded documents without field codes.*

**8-Please provide an example video of the behavioral analysis.**

*R: We now provide an example video as supplemental material.*
